# Vaccination strategy and anti - SARS-CoV-2 S titers in healthcare workers of the INT – IRCCS “Fondazione Pascale” Cancer Center (Naples, Italy)

**DOI:** 10.1186/s13027-021-00375-2

**Published:** 2021-05-12

**Authors:** Ernesta Cavalcanti, Maria Antonietta Isgrò, Domenica Rea, Lucia Di Capua, Giusy Trillò, Luigi Russo, Gerardo Botti, Leonardo Miscio, Franco Maria Buonaguro, Attilio Antonio Montano Bianchi

**Affiliations:** 1 Division of Laboratory Medicine, Istituto Nazionale Tumori – IRCCS Fondazione Pascale, Napoli, Italy; 2 Specialization School in Clinical Pathology and Clinical Biochemistry, School of Medicine and Surgery, Università degli Studi di Napoli Federico II, Napoli, Italy; 3 Istituto Nazionale Tumori – IRCCS Fondazione Pascale, Napoli, Italy

**Keywords:** SARS-CoV-2, Antibody response, Cancer institute, Anti-spike serology, Beads-based linear serology, Anti-SARS-CoV-2 vaccination, Post-COVID-19 single dose vaccine.

## Abstract

**Background:**

Severe Acute Respiratory Syndrome Coronavirus 2 (SARS-CoV-2) infection and the resulting disease, coronavirus disease 2019 (COVID-19), have spread to millions of people globally, requiring the development of billions of different vaccine doses. The SARS-CoV-2 spike mRNA vaccine (named BNT162b2/Pfizer), authorized by the FDA, has shown high efficacy in preventing SARS-CoV-2 infection after administration of two doses in individuals 16 years of age and older.

In the present study, we retrospectively evaluated the differences in the SARS-CoV-2 humoral immune response after vaccine administration in the two different cohorts of workers at the INT - IRCCS “Fondazione Pascale” Cancer Center (Naples, Italy): previously infected to SARS-CoV-2 subjects and not infected to SARS-CoV-2 subjects.

**Methods:**

We determined specific anti-RBD (receptor-binding domain) titers against trimeric spike glycoprotein (S) of SARS-CoV-2 by Roche Elecsys Anti-SARS-CoV-2 S immunoassay in serum samples of 35 healthcare workers with a previous documented history of SARS-CoV-2 infection and 158 healthcare workers without, after 1 and 2 doses of vaccine, respectively. Moreover, geometric mean titers and relative fold changes (FC) were calculated.

**Results:**

Both previously infected and not infected to SARS-CoV-2 subjects developed significant immune responses to SARS-CoV-2 after the administration of 1 and 2 doses of vaccine, respectively. Anti-S antibody responses to the first dose of vaccine were significantly higher in previously SARS-CoV-2-infected subjects in comparison to titers of not infected subjects after the first as well as the second dose of vaccine. Fold changes for subjects previously infected to SARS-CoV-2 was very modest, given the high basal antibody titer, as well as the upper limit of 2500.0 BAU/mL imposed by the Roche methods. Conversely, for naïve subjects, mean fold change following the first dose was low ($\overline{x}$ =1.6), reaching 3.8 FC in 72 subjects (45.6%) following the second dose.

**Conclusions:**

The results showed that, as early as the first dose, SARS-CoV-2-infected individuals developed a remarkable and statistically significant immune response in comparison to those who did not contract the virus previously, suggesting the possibility of administering only one dose in previously SARS-CoV-2-infected subjects. FC for previously infected subjects should not be taken into account for the generally high pre-vaccination values. Conversely, FC for not infected subjects, after the second dose, were = 3.8 in > 45.0% of vaccinees, and ≤ 3.1 in 19.0%, the latter showing a potential susceptibility to further SARS-CoV-2 infection.

## Background

Coronavirus disease 2019 (COVID-19) is a severe acute respiratory syndrome caused by Coronavirus 2 (SARS-CoV-2), a positive-sense single-stranded RNA virus belonging to the *Coronaviridae* family. In 20% of patients the disease evolves to severe pneumonia, respiratory and multi-visceral failure and is responsible for death in patients who present comorbidity, such as diabetes, hypertension, cardiovascular disease and chronic lung disease [1]. The immune system represents an important component against the viral infection through neutralising antibodies production. The trimeric spike glycoprotein (S) of SARS-CoV-2 is a key target for virus neutralising antibodies and the prime candidate for vaccine development [2]. The protein S binds its cellular receptor on the host cells, human angiotensin converting enzyme 2 (ACE), through a receptor-binding domain (RBD) [3]. One of the first vaccines approved by the European Medicines Agency (EMA) was BNT162b2 produced by Pfizer and BioNTech, a vaccine containing the messenger RNA that encodes the SARS-CoV-2 S, in small lipid particles. On 22nd December 2020, the Italian regulatory agency for drugs AIFA (Agenzia Italiana del Farmaco) authorized in Italy the use of BNT162b2/Pfizer vaccine, in 2 doses with an interval of 21 days between the doses [4].

Recent studies have found that subjects infected with COVID-19 present protective immunity for at least 6 months [5], but the impact of previous exposure to SARS-CoV-2 on immune response elicited by the vaccines needs to be verified in a large trial study. Preliminary data reported by Krammer et al., have shown that the immune response to the vaccine after the first dose is substantially more pronounced in individuals with pre-existing immunity and it is similar to the immune response developed after the second dose in individuals not previously infected [6]. Those data have been confirmed in further publications [7, 8]. However, no data are available for people with a history of COVID-19 regarding the booster responses or the ideal dosage, for which reason there is no definitive indication about the administration of the vaccine: whether they should be vaccinated and/or should receive one or two doses [9].

In the present study, we retrospectively analyse antibody responses induced by vaccination in two different cohorts of workers at the INT - IRCCS “Fondazione Pascale” Cancer Center (Naples, Italy): previously infected to SARS-CoV-2 subjects and not infected to SARS-CoV-2 subjects.

## Materials and methods

### Sample size

According to our internal health surveillance program, healthcare workers underwent BNT162b2/Pfizer vaccine: 1 dose was administered to subjects previously infected to SARS-CoV-2 (seropositive for anti-N immunoglobulins), and 2 doses (with an interval of 21 days) to subjects not infected. The program contemplated the evaluation of antibody responses by determining anti-RBD titers at three times: basal, 20 days after the first dose and 8 days after the second dose. Data regarding 193 healthcare workers of INT - IRCCS “Fondazione Pascale” Cancer Centre (35 and 158 with history/no history of COVID-19 infection, defined as infected and not infected to virus subjects, respectively) were collected retrospectively.

### Assay

Roche Elecsys Anti-SARS-CoV-2 S electrochemiluminescence immunoassay (ECLIA) for the in vitro quantitative determination of antibodies (including IgG) against spike RBD of SARS-CoV-2 in human serum was performed on Roche Cobas e 601 module. According to the manufacturer, the correlation test between Roche Elecsys Anti - SARS-CoV-2 S units per mL and WHO International Standards for anti-SARS-CoV-2 immunoglobulins showed an excellent correlation (*r*^2^ = 0.9992, slope = 0.972, intercept = 0.0072), thus allowing to consider specific Roche Elecsys Anti-SARS-CoV-2 S U/mL units equivalent to WHO International Standard BAU/mL (Binding Arbitrary Units per mL). Measuring range spanned from 0.4 BAU/mL to 2500.0 BAU/mL; values higher than 0.8 BAU/mL were considered positive.

### Statistical analysis

Statistical analysis was performed by using the Statistical Package for Social Science (SPSS Inc., Chicago, IL, USA), version 27.0. Distribution of variables was evaluated by Shapiro-Wilk test; parametric data were represented as mean ± standard deviation (SD), whilst non-parametric variables were expressed as median (IR - Interquartile Range). Two-tailed Mann-Whitney (for independent variables) and Wilcoxon (for paired variables) tests were used to compare groups. Values lower than 0.4 BAU/mL were assumed as 0.4 and values higher than 2500.0 BAU/mL were reported as 2500.0; *p* values < 0.05 were considered statistically significant.

To overcome the novelty of general bead-based linear models, used to evaluate post-to-pre vaccination antibody titer increase, the used statistic methods were extrapolated from Zaccaro et al. [10]:

$$ {\log}_{10}(FC)={\log}_{10}\ \left(\frac{z_{28,i}}{z_{0,i}}\right)={\log}_{10}\left({z}_{28,i}\right)-{\log}_{10}\left({z}_{0,i}\right) $$

defining *z*_0, *i*_ and *z*_28, *i*_ to be the Day 0 (pre-vaccination) and Day 28 (post-vaccination) assay results for the i^th^ participant, respectively.

## Results

Data regarding 193 (35 previously infected and 158 not infected healthcare workers) were collected retrospectively: the 35 seropositive cases included 25 female subjects and 10 male subjects with an overall mean age of 48.1 years (SD ± 9.7, range 31–69); the 158 seronegative cases included 74 female subjects and 84 male subjects with an overall mean age of 47.6 years (SD ± 10.0, range 22–70) (Table 1).

**Table 1:** Demographic data of previously infected and not previously infected cohorts of healthcare providers

	Male (age)	Female (age)	Total (mean ± SD)
	n (mean ± SD)		
**Previously infected subjects**	10 (55.2 ± 9.5)	25 (45.2 ± 8.4)	35 (48.1 ± 9.7)
**Not previously infected subjects**	84 (49.7 ± 9.7)	74 (45.2 ± 10.0)	158 (47.6 ± 10.0)
**Total**	94	99	193

Previously infected subjects = subjects infected to SARS-CoV-2

Not previously infected subjects = subjects not infected to SARS-CoV-2

n = number of subjects

mean = mean age

*SD* standard deviation

**Fig. 1:**
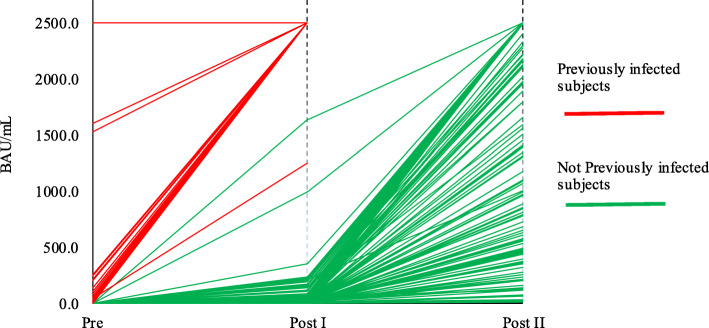
Vaccine immune response monitoring in previously infected and not previously infected cohorts of healthcare providers. Previously infected subjects = subjects previously infected to SARS-CoV-2. Not previously infected subjects = subjects not previously infected to SARS-CoV-2. Pre = anti - SARS-CoV-2 S titers (expressed in BAU/mL) before the first dose. Post I = anti - SARS-CoV-2 S titers (expressed in BAU/mL) 20 days after the first dose. Post II = anti - SARS-CoV-2 S titers (expressed in BAU/mL) 8 days after the second dose

In previously infected to SARS-CoV-2, antibody response 20 days after the first dose of vaccine was statistically higher in comparison to pre-vaccination: median > 2500.0 BAU/mL vs. 36.6 BAU/mL (IR 14.5–99.0) (Wilcoxon test, *p* < 0.001) (Table 2, Fig. 1).

**Table 2:** Vaccine immune response monitoring in previously infected and not previously infected cohorts of healthcare providers

	Pre	Post I	Post II
	Median (IR) BAU/mL		
**Previously infected subjects**	36.6 (14.5–99.0)	> 2500.0	–
**Not previously infected subjects**	< 0.4	18.9 (4.3–58.2)	2111.0 (713.8 - > 2500.0)

Previously infected subjects = subjects infected to SARS-CoV-2

Not previously infected subjects = subjects not infected to SARS-CoV-2

Pre = anti - SARS-CoV-2 S titers before the first dose

Post I = anti - SARS-CoV-2 S titers 20 days after the first dose

Post II = anti - SARS-CoV-2 S titers 8 days after the second dose

median = median of anti - SARS-CoV-2 S titers

IR = Interquartile Range of anti - SARS-CoV-2 S titers

In not infected to SARS-CoV-2 subjects, overall antibody response 20 days after the first dose of vaccine was statistically significant in respect to pre-vaccination values: 18.9 BAU/mL (IR 4.3–58.2) vs. < 0.4 BAU/mL (Wilcoxon test, *p* < 0.001) (Table 2, Fig. 1). Also the titers determined 8 days after the second dose revealed a statistically increasing trend in respect to the first dose: 2111.0 BAU/mL (IR 713.8 - > 2500.0) vs. 18.9 BAU/mL (IR 4.3–58.2) (Wilcoxon test, *p* < 0.001) (Table 2, Fig. 1).

Comparing basal values and titers after the first dose of vaccine in the 2 groups, it was evident that previously infected subjects presented significantly higher basal titers of antibodies in comparison to those not infected (36.6 BAU/mL [IR 14.5–99.0] vs. < 0.4 BAU/mL) and, as early as the first dose, previously infected workers developed a significantly higher antibody response to SARS-CoV-2 respect to values of not infected subjects (> 2500.0 BAU/mL vs. 18.9 BAU/mL [IR 4.3–58.2]), Mann-Whitney test, *p* < 0.001) (Table 2, Fig. 1).

Further interesting information derived from analysis which compared antibody titers after the first dose of vaccine in the previously infected cohort, in comparison to antibody titers after the second dose in the not infected cohort, revealing values significantly higher (> 2500.0 BAU/mL vs. 2111.0 BAU/mL [IR 713.8 - > 2500.0], Mann-Whitney test, *p* < 0.001) (Table 2, Fig. 1).

Fold changes (FC) for subjects previously infected to SARS-CoV-2 was very modest ($\overline{x}$ =1.8) (Table 3), given the high basal antibody titer, as well as the upper limit of 2500.0 BAU/mL imposed by the Roche methods. Conversely, for not infected to SARS-CoV-2 subjects, mean fold change following the first dose was low ($\overline{x}$ =1.6), reaching 3.8 FC in 72 subjects (45.6%) following the second dose (Table 3). In the average, following the second dose, the FC of not infected subjects was 3.5 in comparison to pre-vaccination and 1.9 in comparison to the titer obtained after the first dose of vaccine. In this cohort, given the high variability of FC, prompted us to divide it into 3 different groups, using two arbitrary antibody titers of 500.0 and 800.0 BAU/mL, whose FC values were 3.1 and 3.3, respectively (Table 3). The validation of such arbitrary values will be obtained in the following monitoring of those groups in order to verify and evaluate their relative susceptibility to further SARS-CoV-2 infection.

**Table 3:** Antibody titers’ fold changes (FC) in previously infected and not previously infected cohorts of healthcare providers to monitor vaccine immune responses

Analyzed cohorts	Mean FC	Min FC	Max FC	n (%)
**PreViously infected subjects**				
Post I / Pre	1.8	0.0	3.8	35 (100.0%)
**NotPreviously infected subjects**				
Post I / Pre	1.6	0.0	3.6	158 (100.0%)
Post II / Post I	1.9	0.2	3.8	158 (100.0%)
Post II / Pre	3.5	1.4	3.8	158 (100.0%)
Post II/Pre $\overline{x}$ ≤ 3.1	2.6	1.4	3.1	30 (19.0%)
Post II/Pre 3.1< $\overline{x}$ ≤ 3.3	3.2	3.1	3.3	12 (7.6%)
Post II/Pre 3.3< $\overline{x}$ ≤ 3.8	3.7	3.3	3.8	116 (73.4%)
Post II/Pre = 3.8	3.8			72 (45.6%)

Previously infected subjects = subjects infected to SARS-CoV-2

Not previously infected subjects = subjects not infected to SARS-CoV-2

Pre = anti - SARS-CoV-2 S titers before the first dose

Post I = anti - SARS-CoV-2 S titers 20 days after the first dose

Post II = anti - SARS-CoV-2 S titers 8 days after the second dose

mean FC = mean fold changes of anti - SARS-CoV-2 S mean geometric titers

n (%) = number of subjects (percentage)

## Discussion

Our preliminary data suggest that, differently from behaviour of not previously infected to SARS-CoV-2 subjects, who require 2 doses of vaccine in order to develop a substantial immune response to SARS-CoV-2, antibody titers in previously infected cases present a significant increase since the first dose, as reported for the SARS-CoV-2 infection following the first dose of vaccine. Indeed, the infection would induce a short-lived immunity in some case even “silent” along with a good immune memory, able to trigger a powerful antibody production after an induced stimulus, such as a single vaccine dose. Moreover, although a second dose of vaccine in previously infected subjects will not significantly contribute to their immunization level, it will increase the vaccine reactogenicity [6].

Moreover, these current data suggest that the second dose in not infected subjects reaches the maximal induction in < 50.0% of the vaccinated subjects, with a large variability in the rest of the vaccinees. For such reason, two further subgroups were identified in order to evaluate prospectively their susceptibility to further SARS-CoV-2 infection. It should always be kept in mind that, although we are currently evaluating preferentially the Th2 humoral immune response, most (if not all) anti-SARS-CoV-2 vaccines have been targeted to Th1 immune response [11], and that subjects with low antibody titers could be protected by a strong cellular immunity, which will need to be evaluated in further studies.

## Conclusions

In accordance with first data reported in literature [6, 9], our findings argue that administration of a single dose to previously infected subjects is necessary and that it would be sufficient to elicit an adequate immune response. The vaccine dose serving as booster in naturally infected individuals provides a rationale for updating vaccine recommendations to consider a single vaccine dose to reach protective immunity and to use quantitative anti-RBD serological standardized assays to screen individuals prior to vaccination if the infection history is unknown or uncertain [12, 13].

Nevertheless, further studies are mandatory to confirm these data in larger cohorts of subjects (including patients and immunocompromised individuals) to solve the current gender unbalanced distribution (F to M 2.5:1) and to monitor antibodies’ titers over time. Moreover, more efforts are needed to overcome the use of non-standardized antibodies’ units, the lack of specific cut-offs defining the protective antibody titer levels and the large number of not equivalent diagnostic platforms already commercially available.

## Data Availability

All relevant data and their evaluation are reported in the manuscript.

## Abbreviations

ACE: Human angiotensin converting enzyme 2
AIFA: Agenzia Italiana del Farmaco, the Italian regulatory agency for drugs
BAU/mL: Binding Arbitrary Units per mL
COVID-19: Coronavirus disease 2019
ECLIA: Electrochemiluminescence immunoassay
EMA: European Medicines Agency
FC: Fold Change
RBD: Receptor-binding domain
S: Trimeric spike glycoprotein
SARS-CoV-2: Severe Acute Respiratory Syndrome caused by Coronavirus 2

## References

[1] Zhou F, Yu T, Du R, Fan G, Liu Y, Liu Z, Xiang J, Wang Y, Song B, Gu X (2020). Clinical course and risk factors for mortality of adult inpatients with COVID-19 in Wuhan, China: a retrospective cohort study. Lancet.

[2] Vogel AB, Kanevsky I, Che Y, Swanson KA, Muik A, Vormehr M, et al. BNT162b vaccines protect rhesus macaques from SARS-CoV-2. Nature. 2021. 10.1038/s41586-021-03275-y. 10.1038/s41586-021-03275-y 33524990

[3] Zhou P, Yang XL, Wang XG, Hu B, Zhang L, Zhang W, Si HR, Zhu Y, Li B, Huang CL, Chen HD, Chen J, Luo Y, Guo H, Jiang RD, Liu MQ, Chen Y, Shen XR, Wang X, Zheng XS, Zhao K, Chen QJ, Deng F, Liu LL, Yan B, Zhan FX, Wang YY, Xiao GF, Shi ZL (2020). A pneumonia outbreak associated with a new coronavirus of probable bat origin. Nature.

[4] Filia A, Rota MR, D’Ancona FP (2021). Comirnaty (BNT162b2), Il primo vaccino contro il COVID-19 approvato in Europa e in Italia.

[5] Dan JM, Mateus J, Kato Y, Hastie KM, Yu ED, Faliti CE, Grifoni A, Ramirez SI, Haupt S, Frazier A (2021). Immunological memory to SARS-CoV-2 assessed for up to 8 months after infection. Science.

[6] Krammer F, Srivastava K, Alshammary H, Amoako AA, Awawda MH (2021). Antibody Responses in Seropositive Persons after a Single Dose of SARS-CoV-2 mRNA Vaccine. N Engl J Med.

[7] Padoan A, Dall'Olmo L, Rocca FD, Barbaro F, Cosma C, Basso D, Cattelan A, Cianci V, Plebani M (2021). Antibody response to first and second dose of BNT162b2 in a cohort of characterized healthcare workers. Clin Chim Acta.

[8] Blain H, Tuaillon E, Gamon L, Pisoni A, Miot S, Picot MC, et al. Spike antibody levels of nursing home residents with or without prior COVID-19 3 weeks after a single BNT162b2 vaccine dose. JAMA. 2021. 10.1001/jama.2021.6042 Epub ahead of print. PMID: 33856406. 10.1001/jama.2021.6042 PMC8050783 33856406

[9] Levi R, Azzolini E, Pozzi C, Ubaldi L, Lagioia M, Mantovani A, et al. A cautionary note on recall 1 vaccination in ex-COVID-19 subjects. medRxiv. 2021. 10.1101/202.

[10] Zaccaro DJ, Wagener DK, Whisnant CC, Staats HF (2013). Evaluation of vaccine-induced antibody responses: impact of new technologies. Vaccine.

[11] Sahin U, Muik A, Derhovanessian E, Vogler I, Kranz LM, Vormehr M, Baum A, Pascal K, Quandt J, Maurus D (2020). COVID-19 vaccine BNT162b1 elicits human antibody and TH1 T cell responses. Nature.

[12] Krammer F, Simon V (2020). Serology assays to manage COVID-19. Science..

[13] Amanat F, Stadlbauer D, Strohmeier S, Nguyen THO, Chromikova V, McMahon M, Jiang K, Arunkumar GA, Jurczyszak D, Polanco J, Bermudez-Gonzalez M, Kleiner G, Aydillo T, Miorin L, Fierer DS, Lugo LA, Kojic EM, Stoever J, Liu STH, Cunningham-Rundles C, Felgner PL, Moran T, García-Sastre A, Caplivski D, Cheng AC, Kedzierska K, Vapalahti O, Hepojoki JM, Simon V, Krammer F (2020). A serological assay to detect SARS-CoV-2 seroconversion in humans. Nat Med.

